# Centile reference chart for resting metabolic rate through the life course

**DOI:** 10.1136/archdischild-2022-325249

**Published:** 2023-03-02

**Authors:** Laura Watson, Tim J Cole, Greta Lyons, Christopher Georgiou, Jieniean Worsley, Katherine Carr, Peter Murgatroyd, Carla Moran, Krishna Chatterjee, Michelle Venables

**Affiliations:** 1 NIHR Cambridge Clinical Research Facility, Cambridge, UK; 2 Population Policy and Practice Programme, UCL, London, UK; 3 Metabolic Research Laboratories, Wellcome-MRC Institute of Metabolic Science, Cambridge, UK; 4 Beacon Hospital, University College Dublin School of Medicine, Dublin, Ireland; 5 Stable Isotopes Laboratory, Nutritional Biomarker Laboratory, MRC Epidemiology Unit and Wellcome-MRC Institute of Metabolic Science, Metabolic Research Laboratories, University of Cambridge, Cambridge, UK

**Keywords:** adolescent health, endocrinology, growth, paediatrics, physiology

## Abstract

**Objective:**

Reference centile charts are widely used for the assessment of growth and have progressed from describing height and weight to include body composition variables such as fat and lean mass. Here, we present centile charts for an index of resting energy expenditure (REE) or metabolic rate, adjusted for lean mass versus age, including both children and adults across the life course.

**Design, participants and intervention:**

Measurements of REE by indirect calorimetry and body composition using dual-energy X-ray absorptiometry were made in 411 healthy children and adults (age range 6–64 years) and serially in a patient with resistance to thyroid hormone α (RTHα) between age 15 and 21 years during thyroxine therapy.

**Setting:**

NIHR Cambridge Clinical Research Facility, UK.

**Results:**

The centile chart indicates substantial variability, with the REE index ranging between 0.41 and 0.59 units at age 6 years, and 0.28 and 0.40 units at age 25 years (2nd and 98th centile, respectively). The 50th centile of the index ranged from 0.49 units (age 6 years) to 0.34 units (age 25 years). Over 6 years, the REE index of the patient with RTHα varied from 0.35 units (25th centile) to 0.28 units (<2nd centile), depending on changes in lean mass and adherence to treatment.

**Conclusion:**

We have developed a reference centile chart for an index of resting metabolic rate in childhood and adults, and shown its clinical utility in assessing response to therapy of an endocrine disorder during a patient’s transition from childhood to adult.

**WHAT IS ALREADY KNOWN ON THIS TOPIC?**

- Centile charts for height and weight are widely used in the clinical setting to describe the normality of a measurement.
- Resting energy expenditure (REE) or metabolic rate, being dependent on lean body mass, changes throughout childhood and adult life.

**WHAT THIS STUDY ADDS**

- We have developed a centile chart for REE, based on lean mass and age, across childhood and adult life.
- The centile chart will enable best comparison of this physiological parameter with normality during growth and adolescence, including in response to interventions (eg, thyroid hormone therapy) that govern this variable.

**HOW THIS STUDY MIGHT AFFECT RESEARCH, PRACTICE OR POLICY**

- Centile charts may be most appropriate when comparing other biological parameters that change with human physical growth, with normality.

## Introduction

Describing the physiological phenotypes of uncommon genetic disorders brings the challenge of determining the extent to which characteristics differ from those of a healthy population. This becomes more challenging when the individual is both developing through age and undergoing treatment. Measurement of resting energy expenditure (REE), a physiological variable dependent on body composition, which can change in catabolic (eg, critical illness) or endocrine (eg, hyperthyroidism or hypothyroidism) states, is useful in determining nutritional, energy intake or drug therapy.^1–4^ We have previously reported relationships between REE and body composition, using linear regression modelling and Z-scores, separately in healthy children and adults.^3 5^ As in many other sets of linear prediction equations, REE predictions are not easily interpreted when development moves an individual from one regression segment to another. For example, REE regressions by Schofield^6^ and Henry^7^ which are based on weight and height exhibit this effect. Here, we present an approach to the challenge of describing REE in terms of body composition with a continuous, unsegmented relationship through the 6 to 65 years age range.

Reference, growth centile charts are widely used in paediatric clinical settings to assess measurements such as height, weight and head circumference through childhood.^8^ The current UK-WHO growth charts^9 10^ consist of reference data based on the British 1990 (UK90)^11^ and the WHO standards for weight, length, body mass index (BMI) and head circumference from 2 weeks of age up to 4 years. The UK90 data were also used to construct new charts for birth between 23 and 42 weeks’ gestation.^8^ They are valuable for defining and detecting altered growth in an individual, due to, for example, small for gestational age,^12 13^ childhood obesity,^14^ rapid weight gain,^15^ malnutrition^16^ and epidemiology of childhood health at a population level.^17^

More informatively, body composition reference charts, including data for total body water, fat mass and fat-free mass (FFM) for UK children, adolescents and young adults^18^ and UK infants and children, have emerged.^19^ Body composition can be tracked from infancy to childhood and into adulthood and can be related to many clinical conditions and poor health outcomes. Reference charts allow clinicians to interpret observations across a wide range of diseases, conditions and treatments^20–22^ and are therefore an invaluable tool, contributing to assessment of nutritional status and management in clinical populations.

The advent of genome sequencing,^23^ with earlier diagnosis of heritable metabolic disorders, has enabled commencement of therapies in childhood. Resistance to thyroid hormone alpha (RTHα) or congenital, non-goitrous hypothyroidism 6 (OMIM 614450), due to heterozygous mutations in thyroid hormone receptor α (TRα), is characterised by childhood developmental and growth retardation and a relative hypothyroid state in hormone-resistant TRα-expressing tissues, resulting in reduced REE.^24 25^ Like conventional childhood hypothyroidism, thyroxine therapy alleviates many symptoms and can promote normal growth and development.^2^ However, due to feedback regulation within the pituitary-thyroid axis being mediated by a different, normal thyroid hormone receptor subtype (TRβ), circulating thyroid-stimulating hormone (TSH) is not a useful biomarker in assessing response to thyroxine therapy^2 24 25^

When monitoring therapy of these patients longitudinally, defining expected REE using current methods is difficult. The LMS method fits growth references to data by assuming that the data at each age are distributed as skew normal.^26 27^ The method summarises the reference data with three smooth curves: the power transformation *lambda* needed to normalise the data (the L curve), the median *mu* or 50th centile (M curve), and the coefficient of variation of the distribution *sigma* (the S curve). The three curves allow any required centile to be drawn. The LMS method is a special case of the Generalised Additive Models for Location Scale and Shape (gamlss) family of models^28^ which can be fitted using the gamlss package in R.

The LMS method has been widely used to construct reference data. Wells *et al* 18 19 used it to derive reference centiles for body composition (total body water, FFM and fat mass) and four-component body composition variables from the age of 6 weeks to 20 years. They produced charts with the 25th, 50th, 75th, 91st and 98th centiles.

Using this method, we first sought to construct reference centiles for REE to describe the variation in REE and body composition across childhood and adulthood, providing uniformity across the age spectrum; second, we used the centile chart to plot the change in REE index of a patient with RTHα, evaluating its value in assessing the response to thyroid hormone therapy in this disorder.

## Methods

The REE measurement protocols used have been described previously.^3 5^ All measurements in each participant were made on a single occasion, on waking after an overnight stay in the National Institute for Health and Care Research (NIHR) Cambridge Clinical Research Facility. All investigations were part of ethically approved protocols (RTH: Cambridgeshire LREC 98/154; REC 06/Q0108/84; REC 14/EE/0092) or were clinically indicated and undertaken with prior, informed written consent and assent for children under the age of 16. The healthy participants were free from disease and non-smoking: they were excluded from the study if they were pregnant or receiving any metabolism-influencing medications. Recruitment and data collection took place between 2014 and 2019. Energy expenditure was calculated from the corrected gas exchange volumes (Gem Nutrition, Daresbury, UK) using the derivations of Elia and Livesey.^29^ Body composition was assessed using dual energy X-Ray absorptiometry (DXA, Lunar iDXA, enCORE V.18). Precision of the instruments has been previously reported as iDXA precision for lean mass; 0.4% coefficient of variation (CV), REE precision 3.8% CV.^5 30^

To present the normality of the healthy paediatric (age 6–16 years) data, height, weight and BMI were converted into centiles using the British 1990 WHO reference child data using the excel LMS function.

### Statistical analysis

Statistical analysis was performed using R V.4.0.5 and gamlss package V.5.3-4.

Scatterplots and Pearson’s correlations were examined for all variables (age, height, weight, BMI, bone mass, fat mass and lean mass) considered influential in explaining the variation in REE. Gamlss was used to model the mean and variance of REE as functions of age and body size. Preference was given to log transformed body size variables, to best represent the allometric relationships between them. Age was fitted as a penalised cubic B-spline curve, with age logged to improve the fit by stretching the age range for children relative to adults. Interactions between sex, age and body size were also tested for.

The gamlss package was used to fit the regression models, and the optimal model was identified as that minimising the Bayesian information criterion (BIC). This method has been described elsewhere.^31^

### Centile chart

A centile chart describing the distribution of the REE index (REE/LM^0.67^, kJ/min/kg) was generated from the optimal model, where the centiles were spaced exactly two-thirds of an SD apart, giving the rounded centiles 2, 9, 25, 50, 75, 91 and 98.^32^

## Results

Descriptive characteristics of the healthy children (n=204) and adults (n=126), from which the models were derived, are presented in online supplemental table 1. The participant flow chart is detailed in online supplemental figure 1. The mean BMI SDS for the children in our dataset (aged 6–16.9 years) was 0.41 units for females and 0.27 units for males, when compared with the British 1990-WHO reference child.

10.1136/archdischild-2022-325249.supp1 Supplementary data

The correlations between variables were greater with the variables log-transformed, with the single exception of bone mass versus lean mass (r=0.69 vs 0.76) (online supplemental table 2). Lean mass was the strongest predictor of REE (r=0.77).

The optimal gamlss model predicted log REE as a combination of log lean mass and a spline curve in log age. The coefficient for log lean mass=0.67 SE 0.02, which means that antilogged, the model was equivalent to the index REE/LM^0.67^ (REE index) plotted against age. Adding interaction terms did not reduce the BIC. There was also no evidence of heteroscedasticity—the residual SD was 0.092, corresponding to a residual coefficient of variation in REE of 9.2%, with this residual not depending on age or lean mass.

**Figure 1:**
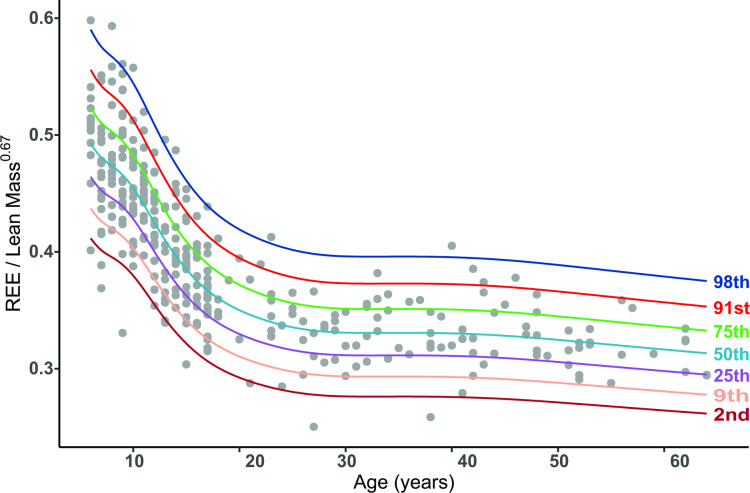
Resting energy expenditure (REE) index centile curves for the 2nd through to the 98th centile plotted against age.

Age-specific centile curves (2nd, 9th, 25th, 50th, 75th, 91st and 98th) and LMS values for the REE index were plotted (figure 1) with the data reported in online supplemental table 3. From age 6 to 25 years, it can be seen that the 50th centile falls by approximately 30% from an REE index of 0.493 units to 0.335 units and then remains relatively constant throughout adulthood. We also observe a large degree of variation within each age, with the 2nd and 98th REE index centiles at age 6 being 0.412 units and 0.590 units, respectively, corresponding to a range of four residual SDs of 9.2%.

**Figure 2:**
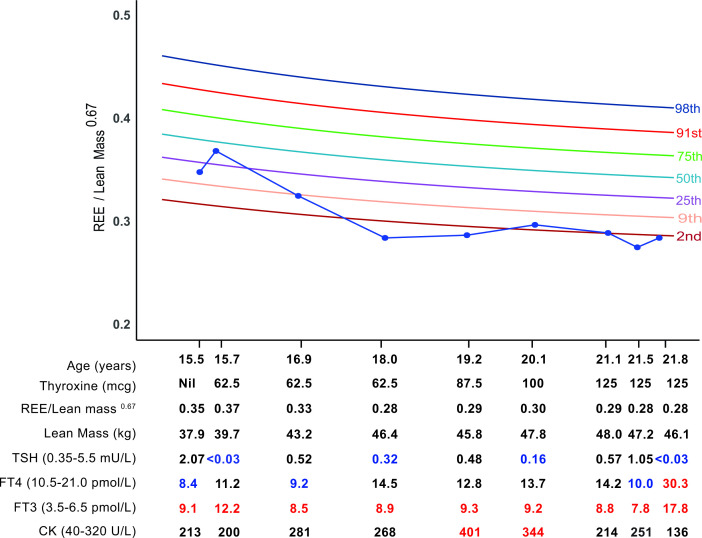
Serial measurements of REE index and biochemical parameters in a patient with RTHα treated with thyroxine therapy over 6 years, superimposed on the centile chart. Biochemical measurements outside the reference range are highlighted blue (low) or red (high). CK, creatine kinase; FT3, free triiodothyronine; FT4, free thyroxine; REE, resting energy expenditure; RTHα, resistance to thyroid hormone α; TSH, thyroid-stimulating hormone.

To illustrate utility of the chart in a clinical setting, serial REE measurements in a patient with RTHα, diagnosed in adolescence and treated with thyroid hormone therapy into adulthood, were superimposed on the centile chart. To monitor the biochemical response to treatment, thyroid function tests (thyroid stimulating hormone (TSH), free thyroxine (FT4), free triiodothyronine (FT3) and creatine kinase (CK), a marker of thyroid hormone action in skeletal muscle) were measured (figure 2).

At baseline (age 15 years), the patient’s REE index was subnormal (REE index 0.348 units, between 9th and 25th centiles) and associated with thyroid biochemistry (low FT4, high FT3, normal TSH), characteristic of RTHα. Initially, thyroxine therapy (62.5 mcg daily) raised circulating FT4 and FT3 concentrations, with a concomitant increase in REE index (0.369 units) of one centile. However, from age 16 to 18 years, his REE index fell sharply (0.284 units, <2nd centile), in association with a marked gain (7 kg) of lean mass that was not matched by an increase in thyroxine dosage, reflected in falling circulating FT3 and rising CK levels. Subsequently, stepwise increases in thyroxine dosage until age 21.1 years, did augment his REE index (0.297 units, above second centile), with concomitant increases in circulating FT3 followed by normalisation of raised CK. At age 21.5 years, a sharp decline in REE index (0.289 to 0.275 units) in the context of stable thyroxine dosage and lean mass suggested that the patient was not adherent with therapy, with biochemical measurements (lower circulating FT4 and FT3, higher CK) confirming this. Latest measurements at age 21.8 years, showing a rise in REE index (0.284 units) and improvement in biochemical indices (higher FT4 and FT3, fall in CK), suggest that adherence with thyroxine therapy has been restored.

## Discussion

We present REE centiles adjusted for lean mass and age over a childhood and adult (age 6 to 65 years) period. Further, we illustrate utility of the REE centile chart in treating a patient with RTHα, a type of congenital hypothyroidism in which this physiological variable best measures the response to thyroxine therapy.

The centile chart consists of the 2nd to the 98th centiles of an REE index, modelled using gamlss. Compared with the UK-WHO reference child, the children in our dataset were mostly representative of a healthy population; however, in 7-year-old girls and 9-year-old boys, the mean centile for BMI was >72. Our results highlight a decline in REE, adjusted for body composition, during childhood and a plateau through adulthood. At each age, we observe a wide degree of variation, with a CV of 9.2%.

Recently, Pontzer *et al*,^33^ reported similar findings to the present study, such that both total energy expenditure (TEE) and basal energy expenditure when adjusted for FFM decline in childhood and reach a plateau at 20 years of age. At 60 years of age, a second break point was apparent when adjusted TEE and basal energy expenditure declined further, potentially linked to a decline in fat mass and FFM.

The reported rapid increase in adjusted TEE during the first year of life has been documented by Reichman *et al,* 34 who have published centile charts for TEE using the LMS method in infants from 1 to 12 months of age. They observed a curvilinear increase in TEE whether expressed as MJ/day or relative to body weight or FFM (MJ/day/kg) throughout this period of rapid growth. Similar to the current study, they observed a large degree of variation at each age and they concluded that a large proportion of the reported variation is biological and not error of measurement.

Our previous linear regression equation for predicting REE in children included lean mass and sex as contributory variables. However, in adults, we and others^3 33 35–37^ have found that fat mass also contributes to variation in REE. In our current work, which combines child and adult datasets (with children comprising 61% of the total), the contribution of fat mass from the adult dataset is insufficient to influence the overall REE index. Whether the centile chart is not appropriate in individuals with grossly deranged fat mass (eg, lipodystrophy) is unclear, but it should be used with caution in such contexts.

We acknowledge that our healthy participant sample is smaller than that recommended for constructing centiles for use at a population level. The WHO growth standards required at least 200 children per sex per 3-month age group for a cross-sectional study.^38^ Other growth reference studies such as the Fourth Dutch Growth Study and the Cuban and First Dutch growth study ranged from 14 500 to 55 000 in sample size.^31^ However, they were focused on height and weight, where the measurements are relatively easy to obtain, whereas capturing REE and lean mass require much more complex technologies. Nevertheless, to address this limitation, we will continue to develop our dataset to close relative paucity of measurements within our sample.

Our application of the centile chart to RTHα, a disorder in which REE is a better marker of thyroid status in TRα-expressing peripheral tissues than circulating TSH measurements,^2^ illustrates its clinical utility. Thus, a decline in REE from age 16 to 18 years accompanied by an increase in lean mass of ~6.7 kg prompted a further increase in dosage of thyroxine therapy to account for growth. Subsequently, a marked fall in REE index (at age 21.5 years) despite stable body composition and thyroxine dosage suggested non-adherence with therapy and biochemical indices (fall in FT4 and FT3) supported this notion. Once non-adherence was rectified, both REE index and biochemical markers improved. Overall, this centile chart enables the impact of both altered body composition and thyroxine therapy on this physiological parameter (REE) to be discerned at the same time. However, when measuring this parameter as a baseline characteristic of RTHα, we suggest that, rather than REE index, deviation of measured REE from a predicted value to derive a Z-score, is a more appropriate but complementary approach.^24^

In conclusion, we have produced an REE index centile chart, adjusted for body composition and age, covering children and adults. This chart enables seamless monitoring of a physiological parameter (REE) that changes with growth, in relation to age-appropriate control data from childhood into adult life. The chart also provides a useful approach to discern the effects of a therapeutic intervention which changes growth, body composition and REE.

## Data Availability

Data are available upon reasonable request. Data described in the manuscript, code book, and analytic code will be made available upon request pending application and approval by contacting lpew2@medschl.cam.ac.uk.

## References

[1] Savage DB , Murgatroyd PR , Chatterjee VK , et al . Energy expenditure and adaptive responses to an acute hypercaloric fat load in humans with lipodystrophy. J Clin Endocrinol Metab 2005;90:1446–52. 10.1210/jc.2004-1494 15613417

[2] Moran C , Agostini M , McGowan A , et al . Contrasting phenotypes in resistance to thyroid hormone alpha correlate with divergent properties of thyroid hormone receptor α1 mutant proteins. Thyroid 2017;27:973–82. 10.1089/thy.2017.0157 28471274 PMC5561448

[3] Watson LPE , Raymond-Barker P , Moran C , et al . An approach to quantifying abnormalities in energy expenditure and lean mass in metabolic disease. Eur J Clin Nutr 2014;68:234–40. 10.1038/ejcn.2013.237 24281313 PMC3916834

[4] Moran C , Habeb AM , Kahaly GJ , et al . Homozygous resistance to thyroid hormone β: can combined antithyroid drug and triiodothyroacetic acid treatment prevent cardiac failure? J Endocr Soc 2017;1:1203–12. 10.1210/js.2017-00204 29264576 PMC5686666

[5] Watson LPE , Carr KS , Venables MC , et al . Quantifying energy expenditure in childhood: utility in managing pediatric metabolic disorders. Am J Clin Nutr 2019;110:1186–91. 10.1093/ajcn/nqz177 31410443 PMC6821543

[6] Schofield WN . Predicting basal metabolic rate, new standards and review of previous work. Hum Nutr Clin Nutr 1985;39 Suppl 1:5–41. 4044297

[7] Henry CJK . Basal metabolic rate studies in humans: measurement and development of new equations. Public Health Nutr 2005;8:1133–52. 10.1079/phn2005801 16277825

[8] Cole TJ , Wright CM , Williams AF , et al . Designing the new UK-WHO growth charts to enhance assessment of growth around birth. Arch Dis Child Fetal Neonatal Ed 2012;97:F219–22. 10.1136/adc.2010.205864 21398325 PMC3546314

[9] de Onis M , Onyango AW , Van den Broeck J , et al . Measurement and standardization protocols for anthropometry used in the construction of a new international growth reference. Food Nutr Bull 2004;25(1_suppl_1):S27–36. 10.1177/15648265040251S105 15069917

[10] Wright CM , Williams AF , Elliman D , et al . Using the new UK-WHO growth charts. BMJ 2010;340:c1140. 10.1136/bmj.c1140 20231247

[11] Cole TJ . The LMS method for constructing normalized growth standards. Eur J Clin Nutr 1990;44:45–60. 2354692

[12] Marcovecchio ML , Gorman S , Watson LPE , et al . Catch-Up growth in children born small for gestational age related to body composition and metabolic risk at six years of age in the UK. Horm Res Paediatr 2020;93:119–27. 10.1159/000508974 32702692

[13] Kaluarachchi DC , Nicksic VB , Allen DB , et al . Thyroid hormone function in small for gestational age term newborns. J Pediatr 2021;238:181–6. 10.1016/j.jpeds.2021.06.067 34214586

[14] Johnson W , Wright J , Cameron N . The risk of obesity by assessing infant growth against the UK-WHO charts compared to the UK90 reference: findings from the born in Bradford birth cohort study. BMC Pediatr 2012;12:104. 10.1186/1471-2431-12-104 22824296 PMC3439315

[15] Lu Y , Pearce A , Li L . Weight gain in early years and subsequent body mass index trajectories across birth weight groups: a prospective longitudinal study. Eur J Public Health 2020;30:316–22. 10.1093/eurpub/ckz232 31899482 PMC7183364

[16] Lara-Pompa NE , Hill S , Williams J , et al . Use of standardized body composition measurements and malnutrition screening tools to detect malnutrition risk and predict clinical outcomes in children with chronic conditions. Am J Clin Nutr 2020;112:1456–67. 10.1093/ajcn/nqaa142 32520318

[17] Group WHOMGRS . Who child growth standards based on length/height, weight and age. Acta Paediatr Suppl 2006;450:76–85. 10.1111/j.1651-2227.2006.tb02378.x 16817681

[18] Wells JCK , Williams JE , Chomtho S , et al . Body-composition reference data for simple and reference techniques and a 4-component model: a new UK reference child. Am J Clin Nutr 2012;96:1316–26. 10.3945/ajcn.112.036970 23076617

[19] Wells JCK , Davies PSW , Fewtrell MS , et al . Body composition reference charts for UK infants and children aged 6 weeks to 5 years based on measurement of total body water by isotope dilution. Eur J Clin Nutr 2020;74:141–8. 10.1038/s41430-019-0409-x 30809008 PMC6949189

[20] Haroun D , Wells JCK , Williams JE , et al . Composition of the fat-free mass in obese and nonobese children: matched case-control analyses. Int J Obes (Lond) 2005;29:29–36. 10.1038/sj.ijo.0802834 15520827

[21] Murphy AJ , Wells JCK , Williams JE , et al . Body composition in children in remission from acute lymphoblastic leukemia. Am J Clin Nutr 2006;83:70–4. 10.1093/ajcn/83.1.70 16400052

[22] Williams JE , Wells JC , Benden C , et al . Body composition assessed by the 4-component model and association with lung function in 6-12-y-old children with cystic fibrosis. Am J Clin Nutr 2010;92:1332–43. 10.3945/ajcn.2010.29847 20926519

[23] Turro E , Astle WJ , Megy K , et al . Whole-Genome sequencing of patients with rare diseases in a national health system. Nature 2020;583:96–102. 10.1038/s41586-020-2434-2 32581362 PMC7610553

[24] Moran C , Agostini M , Visser WE , et al . Resistance to thyroid hormone caused by a mutation in thyroid hormone receptor (TR) α1 and TRα2: clinical, biochemical, and genetic analyses of three related patients. Lancet Diabetes Endocrinol 2014;2:619–26. 10.1016/S2213-8587(14)70111-1 24969835 PMC5989926

[25] Moran C , Chatterjee K . Resistance to thyroid hormone due to defective thyroid receptor alpha. Best Pract Res Clin Endocrinol Metab 2015;29:647–57. 10.1016/j.beem.2015.07.007 26303090 PMC4559105

[26] Cole TJ , Henry CJK . The Oxford Brookes basal metabolic rate database -- a reanalysis. Public Health Nutr 2005;8:1202–12. 10.1079/phn2005806 16277830

[27] Cole TJ , Green PJ . Smoothing reference centile curves: the LMS method and penalized likelihood. Stat Med 1992;11:1305–19. 10.1002/sim.4780111005 1518992

[28] Rigby RA , Stasinopoulos DM . Generalized additive models for location, scale and shape (with discussion). J Royal Statistical Soc C 2005;54:507–54. 10.1111/j.1467-9876.2005.00510.x

[29] Elia M , Livesey G . Energy expenditure and fuel selection in biological systems: the theory and practice of calculations based on indirect calorimetry and tracer methods. World Rev Nutr Diet 1992;70:68–131. 10.1159/000421672 1292242

[30] Watson LPE , Venables MC , Murgatroyd PR . An investigation into the differences in bone density and body composition measurements between 2 ge lunar densitometers and their comparison to a 4-component model. J Clin Densitom 2017;20:498–506. 10.1016/j.jocd.2017.06.029 28756995

[31] Cole TJ . Sample size and sample composition for constructing growth reference centiles. Stat Methods Med Res 2021;30:488–507. 10.1177/0962280220958438 33043801 PMC8008444

[32] Cole TJ . Do growth chart centiles need a face lift? BMJ 1994;308:641–2. 10.1136/bmj.308.6929.641 8148716 PMC2539730

[33] Pontzer H , Yamada Y , Sagayama H , et al . Daily energy expenditure through the human life course. Science 2021;373:808–12. 10.1126/science.abe5017 34385400 PMC8370708

[34] Reichman CA , Davies PSW , Wells JCK , et al . Centile reference charts for total energy expenditure in infants from 1 to 12 months. Eur J Clin Nutr 2003;57:1060–7. 10.1038/sj.ejcn.1601642 12947423

[35] Cunningham JJ . Body composition as a determinant of energy expenditure: a synthetic review and a proposed General prediction equation. Am J Clin Nutr 1991;54:963–9. 10.1093/ajcn/54.6.963 1957828

[36] Nelson KM , Weinsier RL , Long CL , et al . Prediction of resting energy expenditure from fat-free mass and fat mass. Am J Clin Nutr 1992;56:848–56. 10.1093/ajcn/56.5.848 1415003

[37] Nielsen S , Hensrud DD , Romanski S , et al . Body composition and resting energy expenditure in humans: role of fat, fat-free mass and extracellular fluid. Int J Obes Relat Metab Disord 2000;24:1153–7. 10.1038/sj.ijo.0801317 11033984

[38] de Onis M , Garza C , Victora CG , et al . The who multicentre growth reference study: planning, study design, and methodology. Food Nutr Bull 2004;25:S15–26. 10.1177/15648265040251S104 15069916

