# Supplementary material for: Centile reference chart for resting metabolic rate through the life course
Source: Arch Dis Child. 2023 Mar 2;108(7):545–9. doi: 10.1136/archdischild-2022-325249 (PMC7614669; doi:10.1136/archdischild-2022-325249)
Supplement: Supplementary data [file archdischild-2022-325249supp001.pdf]

Appendix Content

Supplementary Table 1. Descriptive characteristics of the healthy participants. **p 2**

Supplementary Table 2. Correlation coefficients for the un-logged and log-transformed variables. **p 3**

Supplementary Table 3. REE index for children and adults by centile, adjusted for lean mass and age. **p 4**

Supplementary Figure 1. Participant flow chart of the study. **p 6**

Watson L, *et al. Arch Dis Child* 2023; 108:545–549. doi: 10.1136/archdischild-2022-325249

Supplementary Table 1. Descriptive characteristics of the healthy participants.

Children	Males (N = 95)		Females (N = 109)	
	Mean ± SD	Range	Mean ± SD	Range
Age (years)	11·2 ± 3·1	6·0 to 16·0	10·5 ± 2·9	6·0 to 16·0
Height (cm)	152·4 ± 20·2	116·8 to 188·2	145·9 ± 17·2	110·7 to 177·2
Weight (kg)	43·7 ± 15·2	20·0 to 78·6	41·1 ± 13·8	18·2 to 84·4
BMI (kg/m^2)	18·2 ± 2·7	14·0 to 29·1	18·7 ± 3·10	13·1 to 28·9
Bone (kg)	1·74 ± 0·70*	0·81 to 3·43	1·53 ± 0·54	0·70 to 2·95
Fat (kg)	9·7 ± 4·2*	3·3 to 26·9	12·3 ± 5·7	4·0 to 32·7
Lean (kg)	32·5 ± 12·3*	16·1 to 62·4	27·4 ± 8·8	13·0 to 49·3
REE (kJ/min)	4·40 ± 0·85*	2·97 to 6·96	3·92 ± 0·64	2·43 to 5·38
**Adults**	**Males (N = 57)**		**Females (N = 69)**	
	**Mean ± SD**	**Range**	**Mean ± SD**	**Range**
Age (years)	31·9 ± 15·0*	17·0 to 64·0	36·2 ± 12·3	17·0 to 62·0
Height (cm)	177·3 ± 7·0	161·8 to 191·1	164·2 ± 7·7	147·0 to 182·9
Weight (kg)	76·0 ± 16·6*	46·6 to 124·0	64·8 ± 12·8	37·7 to 115·0
BMI (kg/m^2)	24·1 ± 4·6	16·7 to 37·2	23·9 ± 3·7	17·4 to 36·3
Bone (kg)	2·96 ± 0·51*	2·06 to 4·20	2·41 ± 0·38	1·53 to 3·32
Fat (kg)	17·5 ± 8·7	5·1 to 43·6	22·1 ± 7·8	6·8 to 48·6
Lean (kg)	56·2 ± 10·2*	36·4 to 91·0	40·7 ± 6·7	24·7 to 64·4
REE (kJ/min)	5·09 ± 0·80*	3·88 to 7·06	3·98 ± 0·56	2·72 to 5·92

Both child and adult datasets have been described in detail previously (3, 5, 29).
*Significant differences between males and females, $P < 0{\cdot}05$.
BMI, Body Mass Index; REE, Resting Energy Expenditure.

Watson L, *et al. Arch Dis Child* 2023; 108:545–549. doi: 10.1136/archdischild-2022-325249

Supplementary Table 2. Correlation coefficients for the un-logged and log-transformed variables.

	Height	Weight	Age	BMI	Bone	Fat	Lean	REE
**Height**	1·00	0·86	0·55	0·53	0·92	0·47	0·91	0·71
**Weight**	0·91	1·00	0·69	0·88	0·92	0·79	0·94	0·71
**Age**	0·71	0·80	1·00	0·65	0·65	0·57	0·62	0·21
**BMI**	0·57	0·86	0·70	1·00	0·69	0·91	0·71	0·51
**Bone**	0·95	0·95	0·79	0·70	1·00	0·55	0·96	0·70
**Fat**	0·57	0·81	0·64	0·90	0·64	1·00	0·53	0·37
**Lean**	0·96	0·96	0·77	0·72	0·97	0·61	1·00	0·78
**REE**	0·72	0·71	0·34	0·51	0·70	0·40	0·77	1·00

Upper triangle unlogged correlations, lower triangle log-transformed correlations.
BMI, Body Mass Index; REE, Resting Energy Expenditure.

Watson L, *et al. Arch Dis Child* 2023; 108:545–549. doi: 10.1136/archdischild-2022-325249

Supplementary Table 3. REE index and LMS values for children and adults by centile, adjusted for lean mass and age.

Age (years)	2nd	9th	25th	50th	75th	91st	98th	Mu	Sigma
**6**	0·412	0·437	0·464	0·493	0·523	0·556	0·590	-0.707	0.090
**7**	0·401	0·426	0·453	0·481	0·510	0·542	0·576	-0.733	0.090
**8**	0·395	0·420	0·446	0·473	0·503	0·534	0·567	-0.748	0.090
**9**	0·389	0·413	0·439	0·466	0·495	0·525	0·558	-0.764	0.090
**10**	0·380	0·403	0·428	0·455	0·483	0·513	0·544	-0.788	0.090
**11**	0·368	0·391	0·415	0·440	0·468	0·497	0·527	-0.820	0.090
**12**	0·355	0·377	0·400	0·425	0·451	0·479	0·509	-0.856	0.090
**13**	0·342	0·364	0·386	0·410	0·435	0·462	0·491	-0.891	0.090
**14**	0·331	0·352	0·374	0·397	0·421	0·447	0·475	-0.925	0.090
**15**	0·322	0·341	0·363	0·385	0·409	0·434	0·461	-0.954	0.090
**16**	0·313	0·333	0·353	0·375	0·398	0·423	0·449	-0.980	0.090
**17**	0·306	0·325	0·346	0·367	0·390	0·414	0·439	-1.003	0.090
**18**	0·301	0·319	0·339	0·360	0·383	0·406	0·431	-1.021	0.090
**19**	0·296	0·315	0·334	0·355	0·377	0·400	0·425	-1.037	0.090
**20**	0·292	0·310	0·330	0·350	0·372	0·395	0·419	-1.050	0.090
**21**	0·289	0·307	0·326	0·346	0·368	0·390	0·414	-1.061	0.090
**22**	0·286	0·304	0·323	0·343	0·364	0·387	0·410	-1.071	0.090
**23**	0·284	0·301	0·320	0·340	0·361	0·383	0·407	-1.079	0.090
**24**	0·282	0·299	0·318	0·337	0·358	0·381	0·404	-1.086	0.090
**25**	0·280	0·297	0·316	0·335	0·356	0·378	0·402	-1.092	0.090
**26**	0·279	0·296	0·314	0·334	0·354	0·376	0·400	-1.097	0.090
**27**	0·278	0·295	0·313	0·333	0·353	0·375	0·398	-1.101	0.090
**28**	0·277	0·294	0·312	0·332	0·352	0·374	0·397	-1.104	0.090
**29**	0·277	0·294	0·312	0·331	0·352	0·373	0·397	-1.105	0.090
**30**	0·276	0·293	0·312	0·331	0·351	0·373	0·396	-1.106	0.090
**31**	0·276	0·293	0·311	0·331	0·351	0·373	0·396	-1.107	0.090
**32**	0·276	0·293	0·311	0·331	0·351	0·373	0·396	-1.107	0.090
**33**	0·276	0·293	0·311	0·331	0·351	0·373	0·396	-1.107	0.090
**34**	0·276	0·293	0·311	0·331	0·351	0·373	0·396	-1.107	0.090
**35**	0·276	0·293	0·311	0·331	0·351	0·373	0·396	-1.107	0.090
**36**	0·276	0·293	0·311	0·331	0·351	0·373	0·396	-1.107	0.090

Watson L, *et al. Arch Dis Child* 2023; 108:545–549. doi: 10.1136/archdischild-2022-325249

**37**	0·276	0·293	0·311	0·331	0·351	0·373	0·396	-1.107	0.090
**38**	0·276	0·293	0·311	0·331	0·351	0·373	0·396	-1.107	0.090
**39**	0·276	0·293	0·311	0·330	0·351	0·373	0·396	-1.108	0.090
**40**	0·276	0·293	0·311	0·330	0·351	0·372	0·395	-1.108	0.090
**41**	0·276	0·293	0·311	0·330	0·350	0·372	0·395	-1.109	0.090
**42**	0·275	0·292	0·310	0·330	0·350	0·372	0·395	-1.110	0.090
**43**	0·275	0·292	0·310	0·329	0·350	0·371	0·394	-1.111	0.090
**44**	0·275	0·291	0·310	0·329	0·349	0·371	0·394	-1.113	0.090
**45**	0·274	0·291	0·309	0·328	0·348	0·370	0·393	-1.114	0.090
**46**	0·274	0·290	0·308	0·328	0·348	0·369	0·392	-1.116	0.090
**47**	0·273	0·290	0·308	0·327	0·347	0·369	0·391	-1.118	0.090
**48**	0·272	0·289	0·307	0·326	0·346	0·368	0·391	-1.120	0.090
**49**	0·272	0·289	0·306	0·325	0·346	0·367	0·390	-1.123	0.090
**50**	0·271	0·288	0·306	0·325	0·345	0·366	0·389	-1.125	0.090
**51**	0·270	0·287	0·305	0·324	0·344	0·365	0·388	-1.127	0.090
**52**	0·270	0·286	0·304	0·323	0·343	0·364	0·387	-1.130	0.090
**53**	0·269	0·286	0·303	0·322	0·342	0·363	0·386	-1.133	0.090
**54**	0·268	0·285	0·303	0·321	0·341	0·362	0·385	-1.135	0.090
**55**	0·268	0·284	0·302	0·321	0·340	0·361	0·384	-1.138	0.090
**56**	0·267	0·284	0·301	0·320	0·339	0·361	0·383	-1.140	0.090
**57**	0·266	0·283	0·300	0·319	0·339	0·360	0·382	-1.143	0.090
**58**	0·266	0·282	0·299	0·318	0·338	0·359	0·381	-1.146	0.090
**59**	0·265	0·281	0·299	0·317	0·337	0·358	0·380	-1.148	0.090
**60**	0·264	0·281	0·298	0·316	0·336	0·357	0·379	-1.151	0.090
**61**	0·264	0·280	0·297	0·316	0·335	0·356	0·378	-1.153	0.090
**62**	0·263	0·279	0·296	0·315	0·334	0·355	0·377	-1.156	0.090
**63**	0·262	0·278	0·296	0·314	0·333	0·354	0·376	-1.158	0.090
**64**	0·262	0·278	0·295	0·313	0·333	0·353	0·375	-1.161	0.090

Watson L, *et al. Arch Dis Child* 2023; 108:545–549. doi: 10.1136/archdischild-2022-325249

Supplementary Figure 1. Participant flow chart of the study.

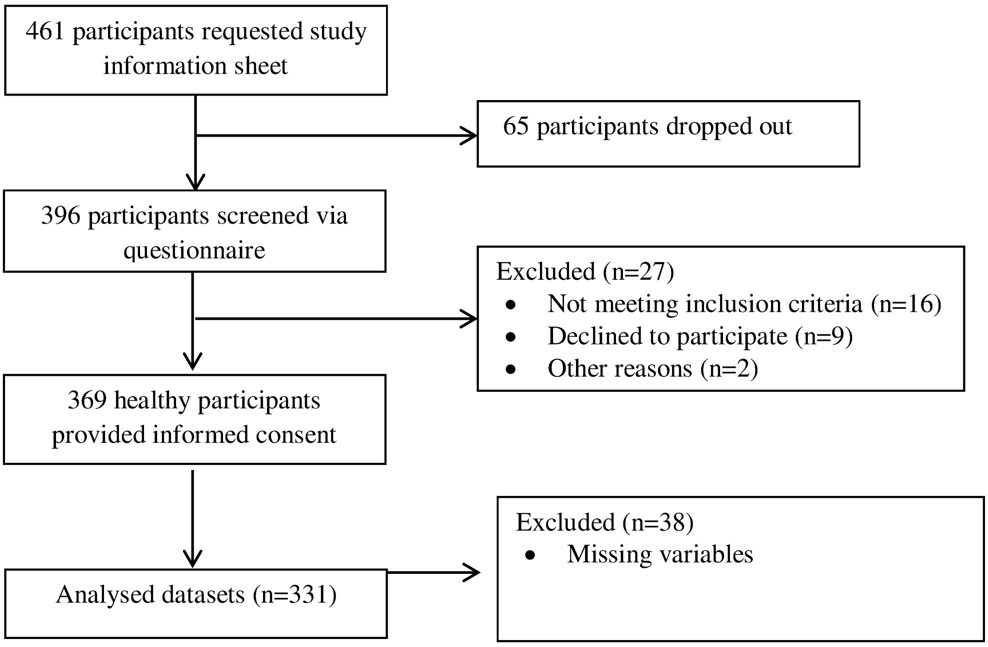

Watson L, *et al. Arch Dis Child* 2023; 108:545–549. doi: 10.1136/archdischild-2022-325249
