# Supplementary material for: Centile reference chart for resting metabolic rate through the life course
Source: Arch Dis Child. Author manuscript; Available in PMC 2023 Jul 1. (PMC7614669; doi:10.1136/archdischild-2022-325249)

Appendix Content

Supplementary Table 1. Descriptive characteristics of the healthy participants. **p 2**

Supplementary Table 2. Correlation coefficients for the un-logged and log-transformed variables. **p 3**

Supplementary Table 3. REE index for children and adults by centile, adjusted for lean mass and age. **p 4**

Supplementary Figure 1. Participant flow chart of the study. **p 6**

Supplementary Table 1. Descriptive characteristics of the healthy participants.

Children	Males (N = 95)		Females (N = 109)	
	**Mean ± SD**	**Range**	**Mean ± SD**	**Range**
**Age (years)**	11·2 ± 3·1	6·0 to 16·0	10·5 ± 2·9	6·0 to 16·0
**Height (cm)**	152·4 ± 20·2	116·8 to 188·2	145·9 ± 17·2	110·7 to 177·2
**Weight (kg)**	43·7 ± 15·2	20·0 to 78·6	41·1 ± 13·8	18·2 to 84·4
**BMI (kg/m^2)**	18·2 ± 2·7	14·0 to 29·1	18·7 ± 3·10	13·1 to 28·9
**Bone (kg)**	1·74 ± 0·70*	0·81 to 3·43	1·53 ± 0·54	0·70 to 2·95
**Fat (kg)**	9·7 ± 4·2*	3·3 to 26·9	12·3 ± 5·7	4·0 to 32·7
**Lean (kg)**	32·5 ± 12·3*	16·1 to 62·4	27·4 ± 8·8	13·0 to 49·3
**REE (kJ/min)**	4·40 ± 0·85*	2·97 to 6·96	3·92 ± 0·64	2·43 to 5·38
**Adults**	**Males (N = 57)**		**Females (N = 69)**	
	**Mean ± SD**	**Range**	**Mean ± SD**	**Range**
**Age (years)**	31·9 ± 15·0*	17·0 to 64·0	36·2 ± 12·3	17·0 to 62·0
**Height (cm)**	177·3 ± 7·0	161·8 to 191·1	164·2 ± 7·7	147·0 to 182·9
**Weight (kg)**	76·0 ± 16·6*	46·6 to 124·0	64·8 ± 12·8	37·7 to 115·0
**BMI (kg/m^2)**	24·1 ± 4·6	16·7 to 37·2	23·9 ± 3·7	17·4 to 36·3
**Bone (kg)**	2·96 ± 0·51*	2·06 to 4·20	2·41 ± 0·38	1·53 to 3·32
**Fat (kg)**	17·5 ± 8·7	5·1 to 43·6	22·1 ± 7·8	6·8 to 48·6
**Lean (kg)**	56·2 ± 10·2*	36·4 to 91·0	40·7 ± 6·7	24·7 to 64·4
**REE (kJ/min)**	5·09 ± 0·80*	3·88 to 7·06	3·98 ± 0·56	2·72 to 5·92

Both child and adult datasets have been described in detail previously (3, 5, 29).
*Significant differences between males and females, $P < 0·05$.
BMI, Body Mass Index; REE, Resting Energy Expenditure.

Supplementary Table 2. Correlation coefficients for the un-logged and log-transformed variables.

	**Height**	**Weight**	**Age**	**BMI**	**Bone**	**Fat**	**Lean**	**REE**
**Height**	1·00	0·86	0·55	0·53	0·92	0·47	0·91	0·71
**Weight**	0·91	1·00	0·69	0·88	0·92	0·79	0·94	0·71
**Age**	0·71	0·80	1·00	0·65	0·65	0·57	0·62	0·21
**BMI**	0·57	0·86	0·70	1·00	0·69	0·91	0·71	0·51
**Bone**	0·95	0·95	0·79	0·70	1·00	0·55	0·96	0·70
**Fat**	0·57	0·81	0·64	0·90	0·64	1·00	0·53	0·37
**Lean**	0·96	0·96	0·77	0·72	0·97	0·61	1·00	0·78
**REE**	0·72	0·71	0·34	0·51	0·70	0·40	0·77	1·00

Upper triangle unlogged correlations, lower triangle log-transformed correlations.
BMI, Body Mass Index; REE, Resting Energy Expenditure.

Supplementary Table 3. REE index and LMS values for children and adults by centile, adjusted for lean mass and age.

Age (years)	2nd	9th	25th	50th	75th	91st	98th	Mu	Sigma
**6**	0·412	0·437	0·464	0·493	0·523	0·556	0·590	-0.707	0.090
**7**	0·401	0·426	0·453	0·481	0·510	0·542	0·576	-0.733	0.090
**8**	0·395	0·420	0·446	0·473	0·503	0·534	0·567	-0.748	0.090
**9**	0·389	0·413	0·439	0·466	0·495	0·525	0·558	-0.764	0.090
**10**	0·380	0·403	0·428	0·455	0·483	0·513	0·544	-0.788	0.090
**11**	0·368	0·391	0·415	0·440	0·468	0·497	0·527	-0.820	0.090
**12**	0·355	0·377	0·400	0·425	0·451	0·479	0·509	-0.856	0.090
**13**	0·342	0·364	0·386	0·410	0·435	0·462	0·491	-0.891	0.090
**14**	0·331	0·352	0·374	0·397	0·421	0·447	0·475	-0.925	0.090
**15**	0·322	0·341	0·363	0·385	0·409	0·434	0·461	-0.954	0.090
**16**	0·313	0·333	0·353	0·375	0·398	0·423	0·449	-0.980	0.090
**17**	0·306	0·325	0·346	0·367	0·390	0·414	0·439	-1.003	0.090
**18**	0·301	0·319	0·339	0·360	0·383	0·406	0·431	-1.021	0.090
**19**	0·296	0·315	0·334	0·355	0·377	0·400	0·425	-1.037	0.090
**20**	0·292	0·310	0·330	0·350	0·372	0·395	0·419	-1.050	0.090
**21**	0·289	0·307	0·326	0·346	0·368	0·390	0·414	-1.061	0.090
**22**	0·286	0·304	0·323	0·343	0·364	0·387	0·410	-1.071	0.090
**23**	0·284	0·301	0·320	0·340	0·361	0·383	0·407	-1.079	0.090
**24**	0·282	0·299	0·318	0·337	0·358	0·381	0·404	-1.086	0.090
**25**	0·280	0·297	0·316	0·335	0·356	0·378	0·402	-1.092	0.090
**26**	0·279	0·296	0·314	0·334	0·354	0·376	0·400	-1.097	0.090
**27**	0·278	0·295	0·313	0·333	0·353	0·375	0·398	-1.101	0.090
**28**	0·277	0·294	0·312	0·332	0·352	0·374	0·397	-1.104	0.090
**29**	0·277	0·294	0·312	0·331	0·352	0·373	0·397	-1.105	0.090
**30**	0·276	0·293	0·312	0·331	0·351	0·373	0·396	-1.106	0.090
**31**	0·276	0·293	0·311	0·331	0·351	0·373	0·396	-1.107	0.090
**32**	0·276	0·293	0·311	0·331	0·351	0·373	0·396	-1.107	0.090
**33**	0·276	0·293	0·311	0·331	0·351	0·373	0·396	-1.107	0.090
**34**	0·276	0·293	0·311	0·331	0·351	0·373	0·396	-1.107	0.090
**35**	0·276	0·293	0·311	0·331	0·351	0·373	0·396	-1.107	0.090
**36**	0·276	0·293	0·311	0·331	0·351	0·373	0·396	-1.107	0.090

**37**	0·276	0·293	0·311	0·331	0·351	0·373	0·396	-1.107	0.090
**38**	0·276	0·293	0·311	0·331	0·351	0·373	0·396	-1.107	0.090
**39**	0·276	0·293	0·311	0·330	0·351	0·373	0·396	-1.108	0.090
**40**	0·276	0·293	0·311	0·330	0·351	0·372	0·395	-1.108	0.090
**41**	0·276	0·293	0·311	0·330	0·350	0·372	0·395	-1.109	0.090
**42**	0·275	0·292	0·310	0·330	0·350	0·372	0·395	-1.110	0.090
**43**	0·275	0·292	0·310	0·329	0·350	0·371	0·394	-1.111	0.090
**44**	0·275	0·291	0·310	0·329	0·349	0·371	0·394	-1.113	0.090
**45**	0·274	0·291	0·309	0·328	0·348	0·370	0·393	-1.114	0.090
**46**	0·274	0·290	0·308	0·328	0·348	0·369	0·392	-1.116	0.090
**47**	0·273	0·290	0·308	0·327	0·347	0·369	0·391	-1.118	0.090
**48**	0·272	0·289	0·307	0·326	0·346	0·368	0·391	-1.120	0.090
**49**	0·272	0·289	0·306	0·325	0·346	0·367	0·390	-1.123	0.090
**50**	0·271	0·288	0·306	0·325	0·345	0·366	0·389	-1.125	0.090
**51**	0·270	0·287	0·305	0·324	0·344	0·365	0·388	-1.127	0.090
**52**	0·270	0·286	0·304	0·323	0·343	0·364	0·387	-1.130	0.090
**53**	0·269	0·286	0·303	0·322	0·342	0·363	0·386	-1.133	0.090
**54**	0·268	0·285	0·303	0·321	0·341	0·362	0·385	-1.135	0.090
**55**	0·268	0·284	0·302	0·321	0·340	0·361	0·384	-1.138	0.090
**56**	0·267	0·284	0·301	0·320	0·339	0·361	0·383	-1.140	0.090
**57**	0·266	0·283	0·300	0·319	0·339	0·360	0·382	-1.143	0.090
**58**	0·266	0·282	0·299	0·318	0·338	0·359	0·381	-1.146	0.090
**59**	0·265	0·281	0·299	0·317	0·337	0·358	0·380	-1.148	0.090
**60**	0·264	0·281	0·298	0·316	0·336	0·357	0·379	-1.151	0.090
**61**	0·264	0·280	0·297	0·316	0·335	0·356	0·378	-1.153	0.090
**62**	0·263	0·279	0·296	0·315	0·334	0·355	0·377	-1.156	0.090
**63**	0·262	0·278	0·296	0·314	0·333	0·354	0·376	-1.158	0.090
**64**	0·262	0·278	0·295	0·313	0·333	0·353	0·375	-1.161	0.090

Supplementary Figure 1. Participant flow chart of the study.

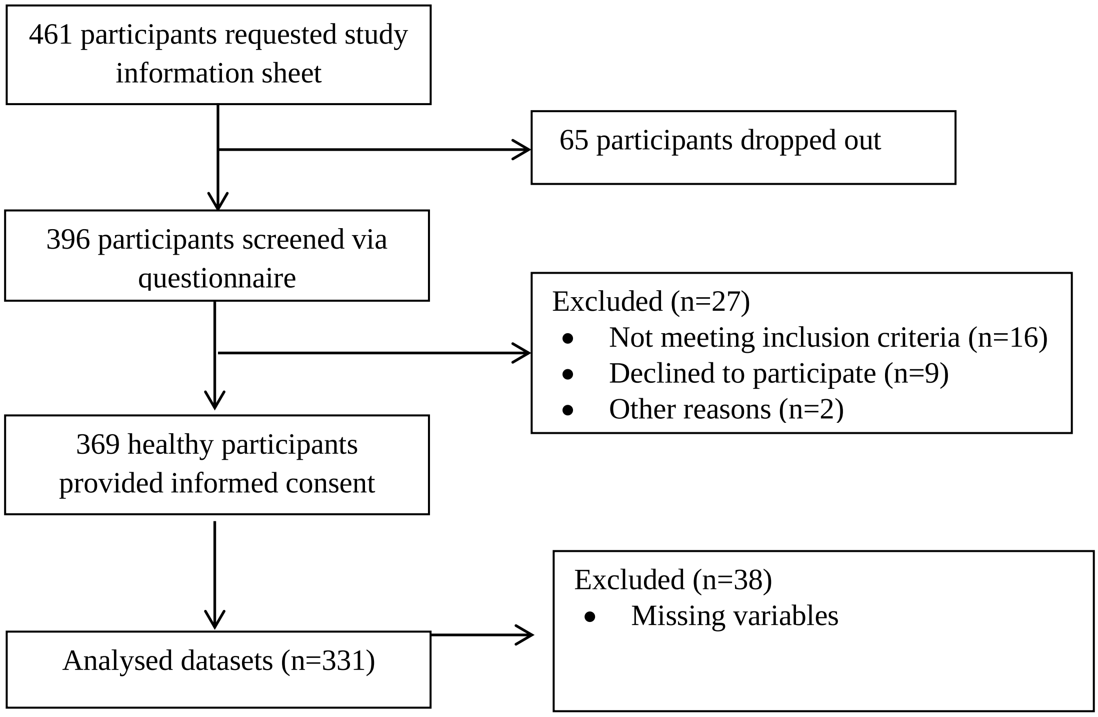

Supplement: Supplementary file 1 [file EMS172179-supplement-Appendix_Content.pdf]
